# Construction and validation of a fatty acid metabolism-related gene signature for predicting prognosis and therapeutic response in patients with prostate cancer

**DOI:** 10.7717/peerj.14854

**Published:** 2023-02-06

**Authors:** Hongjun Zhao, Tong Wu, Zehao Luo, Qinyao Huang, Sihua Zhu, Chunling Li, Zubing Zhang, Jiahao Zhang, Jianwen Zeng, Yuying Zhang

**Affiliations:** 1 Department of Urology, The Sixth Affiliated Hospital of Guangzhou Medical University, Qingyuan, China; 2 Shenzhen Longhua Maternity and Child Healthcare Hospital, Shenzhen, China

**Keywords:** Prostate cancer, Prognosis, Fatty acid metabolism, Tumorigenesis

## Abstract

**Background:**

Reprogramming of fatty acid metabolism is a newly-identified hallmark of malignancy. However, no studies have systematically investigated the fatty acid metabolism related-gene set in prostate cancer (PCa).

**Methods:**

A cohort of 381 patients with gene expression and clinical data from The Cancer Genome Atlas was used as the training set, while another cohort of 90 patients with PCa from GEO (GSE70769) was used as the validation set. Differentially expressed fatty acid metabolism-related genes were subjected to least absolute shrinkage and selection operator (LASSO)-Cox regression to establish a fatty acid metabolism-related risk score. Associations between the risk score and clinical characteristics, immune cell infiltration, tumor mutation burden (TMB), tumor immune dysfunction and exclusion (TIDE) score, and response to chemotherapy were analyzed. Finally, the expression level of genes included in the model was validated using real-time PCR.

**Results:**

A prognostic risk model based on five fatty acid metabolism related genes (*ALDH1A1*, *CPT1B*, *CA2*, *CROT*, and *NUDT19*) were constructed. Tumors with higher risk score were associated with larger tumor size, lymph node involvement, higher Gleason score, and poorer biochemical recurrence (BCR)-free survival. Furthermore, the high- and low-risk tumors exhibited distinct immune cell infiltration features and immune-related pathway activation. High-risk tumors were associated with favorable response to immunotherapy as indicated by high TMB and low TIDE score, but poor response to bicalutamide and docetaxel chemotherapy.

**Conclusion:**

This study established a fatty acid metabolism-related gene signature which was predictive of BCR and response to chemotherapy and immunotherapy, providing a novel therapeutic biomarker for PCa.

## Introduction

Prostate cancer (PCa) is one of the most common malignancies and the fifth leading cause of cancer-related death in men worldwide (Sung et al., 2021). Although the prognosis of prostate cancer has greatly improved during the past decades (Faria et al., 2020; Hapuarachchige & Artemov, 2020; Moore, 2020; Song et al., 2020), survival rate and quality of life will decrease once biochemical recurrence (BCR) occurs (May et al., 2016). Unfortunately, repeated BCRs cannot be avoided despite aggressive treatment (Zumsteg et al., 2015). Therefore, prognostic predictors of BCR are needed for risk stratification and personalized clinical decision making. However, those widely used prognostic factors, such as pre- and post-treatment prostate-specific antigen (PSA) value, Gleason score, pathological tumor size, cannot effectively identify patients at high risk of recurrence. Therefore, there is an unmet need of effective prognostic biomarkers for PCa to facilitate personalized therapy and improve prognosis.

Metabolic reprogramming is a well-recognized hallmark of malignancy and plays a crucial role in tumorigenesis (Hanahan & Weinberg, 2011). Reprogramming of lipid metabolism is among the most significant metabolic changes that enable cancer cells to survive and thrive in the harsh tumor microenvironment (TME). Increased lipid uptake, storage, and lipogenesis commonly occur in malignant tumors and are closely related to tumor initiation and progression (Cheng et al., 2018). As an important component of lipids, fatty acid metabolism plays an important role in energy storage, biofilm synthesis, and production of signaling molecules involving tumorigenesis (Currie et al., 2013). Increased fatty acid synthase levels confer proliferative and invasive advantages (De Piano et al., 2021), and suppressing fatty acid uptake was reported to slow PCa progression (Watt et al., 2019). Thus, targeting at fatty acid metabolic pathways might be a promising therapeutic strategy for PCa.

Metabolic profiling analysis might enhance our understanding on the molecular mechanisms and provide further guidance on the risk stratification and individualized therapy for tumors. For example, a prognostic risk signature based on eight fatty acid metabolism-related genes was developed in glioma, and it was found to be closely related to glioma prognosis and immune cell infiltration features in the TME (Qi et al., 2019). More recently, Su et al. (2022) constructed and validated a metabolic-related gene signature which was able to predict BCR in primary PCa. However, the significance of a fatty acid metabolism-related gene set in PCa remains unclear.

In this study, using data from The Cancer Genome Atlas (TCGA) dataset and the GEO dataset (GSE70769), we developed a prognostic risk signature for PCa based on fatty-acid metabolism-related genes. This risk signature demonstrated a robust prognosis predictive ability and was closely associated with tumor immune cell infiltration features and immune-related pathway activation. Moreover, high risk tumors exhibited a potentially favorable response to immunotherapy and unfavorable response to chemotherapy. Finally, we validated the gene expression of the prognostic fatty-acid metabolism-related genes in cells. Our results provide insight into the molecular mechanisms of PCa and also provide a novel tool for treatment response prediction and clinical risk stratification.

## Materials and Methods

### Patients and datasets

RNA-seq (FPKM value) and clinical data from TCGA were obtained from the UCSC Xena website (https://xena.ucsc.edu/welcome-to-ucsc-xena), including 381 PCa and 52 normal prostate sample data. Raw data were transformed into transcripts per kilobase million (TPM) values and log2(TPM + 1) transformed for subsequent analysis. The GSE70769 dataset downloaded from the Gene Expression Omnibus (GEO) dataset (https://www.ncbi.nlm.nih.gov/geo/), containing the genomic profile and clinical data of 90 prostate tumors, was used as the validation cohort. Patients with incomplete information on BCR in the TCGA and GEO databases were excluded. The endpoint BCR was defined as two consecutive PSA values of ≥0.2 ng/ml (Heidenreich et al., 2008).

### Fatty acid metabolism-related genes

Three gene sets related to fatty acid metabolism—Hallmark fatty acid metabolism genes, Kyoto Encyclopedia of Genes and Genomes (KEGG) fatty acid metabolism pathways, and Reactome fatty acid metabolism genes—were extracted from the Molecular Signature Database v7.5.1 (MSigDB; https://www.gsea-msigdb.org/gsea/msigdb), and 309 fatty acid metabolism-related genes were retrieved after removing overlapping genes (Table S1). One of the fatty acid metabolism-related genes (*XIST*) was unavailable in the TCGA, leaving 308 genes for further analyses.

### Identification of differentially expressed genes (DEGs)

DEGs were identified by comparing the mRNA expression of 308 fatty acid related genes between tumor and normal tissue in TCGA datasets using the “limma” package. Genes with a false discovery rate (FDR) <0.05 and |log FC| >0.5 were selected for further analysis. DEGs between the low- and high-risk groups were identified in TCGA after the calculation of the risk score using the “limma” package. Genes with adjusted FDR <0.05 and |log FC| >0.5 were selected for further analysis.

### Establishment and validation of the risk predictive model

Univariate Cox regression analysis was used to determine the fatty acid-related genes whose expression levels were significantly correlated with BCR-free survival in patients with PCa (*P* < 0.05). The least absolute shrinkage and selection operator (LASSO) Cox regression method was applied to these genes. Finally, multiple stepwise Cox regression, with both backward and forward selection, was performed on the candidate fatty acid-related genes, and the prognostic model was determined based on the lowest Akaike information criterion (AIC) value. The fatty acid-related gene score was calculated based on the gene expression level and the corresponding regression coefficient. The formula was established as follows: Risk Score = expression level of gene1*Coef1 + expression level of gene2*Coef2 + … + expression level of genex*Coefx (Jiang et al., 2022). The median score was used as the cut-off value to divide TCGA patients with PCa into high- and low-risk groups. The “survival”, “glmnet” and “survminer” R package were used to establish the risk predictive model. The same formula was applied to the GEO dataset for validation purposes.

Univariate and multivariate Cox proportional hazards regression analyses were performed to test whether the risk score was an independent prognostic factor in the TCGA and GEO databases. The Kaplan–Meier (KM) survival curve was used to compare the BCR-free survival of patients in the high- and low-risk groups. Principal component analysis (PCA) and t-distributed stochastic neighbor embedding (t-SNE) were used to explore the distribution of different populations. Time-dependent receiver operating curve (ROC) curve analysis was used to evaluate the predictive power of the gene signatures and clinicopathological features.

### Functional enrichment analysis

The list of DEGs between tumor and normal tissues in the TCGA dataset was subjected to the Metascape online tool (https://metascape.org/) for functional enrichment analysis. The “clusterProfiler” R package was used to perform Gene Ontology (GO) and KEGG analyses based on the DEGs between different risk groups. The infiltration scores of 28 immune cells and the activities of 13 immune-related pathways were calculated using the “GSVA” R package by single-sample gene set enrichment analysis (ssGSEA).

### Prediction of responses to immunotherapy and chemotherapy

Tumor immune dysfunction and exclusion (TIDE) score and tumor mutation burden (TMB) were used to assess the clinical response to immune therapy in patients with PCa. A low TIDE score suggested high sensitivity to immunotherapy (Jiang et al., 2018), while a high TMB was suggestive of high sensitivity to immunotherapy (Yarchoan, Hopkins & Jaffee, 2017). The TIDE score was calculated according to algorithm proposed by Jiang et al. (2018) in a previous study. We obtained the TCGA mutation dataset from the TCGA website (https://portal.gdc.cancer.gov/repository), and each patient’s TMB was measured as the total number of non-synonymous mutations per megabase. In addition, chemotherapeutic response to three commonly used drugs (bicalutamide, cisplatin, and docetaxel) for patients with PCa in the TCGA dataset was calculated using the “pRRophetic” R package.

### Cell culture

The normal prostate epithelial (RWPE-1) cells (Cellcook, Guangzhou, China) were cultured in Keratinocyte SFM medium (Sciencell, Carlsbad, CA, USA) containing 1% bovine pituitary extract (M&C Gene, Beijing, China) and 1% epidermal growth factor (Sciencell, Carlsbad, CA, USA). PC3 and LNCaP cells (Cellcook, Guangzhou, China) were cultured in RPMI-1640 medium (Gibco, Waltham, MA, USA) containing 10% fetal bovine serum (Bioind, Cromwell, CT, USA). All cells were cultured in a water-saturated atmosphere with 5% CO_2_ at 37 °C.

### RNA isolation and real-time PCR

Total RNA was extracted using TRIzol reagent according to the manufacturer’s instructions. The concentration and purity of all the RNA samples were determined at an absorbance ratio of 260/280 nm. The PrimeScript RT Reagent Kit with gDNA Eraser (Takara, San Jose, CA, USA) was used for cDNA synthesis. Quantitative real-time PCR (qPCR) using the Fast SYBR Green PCR Master Mix (Applied Biosystems, Waltham, MA, USA) was performed on a StepOne Real-Time PCR System (Applied Biosystems, Waltham, MA, USA). The relative level of mRNA expression of a gene was determined by normalization to GAPDH. The 2^−ΔΔCT^ method was used for data analysis. The primers used for real-time PCR were as follows:

ALDH1A1-F: 5′-CCGTGGCGTACTATGGATGC-3′;

ALDH1A1-R: 5′-GCAGCAGACGATCTCTTTCGAT-3′;

CA2-F: 5′-AAGGAACCCATCAGCGTCAG-3′;

CA2-R: 5′-TTCTTCAGTGGCTGAGCTGG-3′;

CPT1B-F: 5′-CCTGCTACATGGCAACTGCTA-3′;

CPT1B-R: 5′-AGAGGTGCCCAATGATGGGA′;

CROT-F: 5′-ATTGGCTGGAAGAGTGGTGG-3′;

CROT-R: 5′-GAGTCCCTTCCTTTGGAGGC-3′;

NUDT19-F: 5′-CCCCACAGTTCTACGAAGTGA-3′;

NUDT19-R: 5′-TCTAATGCACGACCCAAACAAA-3′;

### Statistical analysis

All data analyses were performed using R (v.4.1.3; R Core Team, 2022).

A Student’s t-test was used to measure differential expression of ALDH1A1, CA2, CPT1B, CROT and NUDT19 in subgroups, including in two prostate cancer cell lines (PC3 and LNCAP) and a normal prostate cell line (RWPE-1) and between tumor samples and normal samples in the TCGA cohort. The Wilcoxon test and Kruskal–Wallis test were used for between-group comparisons, including risk score, patient age, Gleason score, PSA value, tumor pathological T-stage, and N-stage in the TCGA cohort. Kaplan–Meier survival analysis and log-rank test were used to compare the differences in BCR-free survival between the stratified groups. Univariate and multivariate Cox regression analyses were used to determine the independent prognostic factors. Statistical significance was set at *P* < 0.05.

## Results

### Identification of fatty acid-related DEGs and functional analysis

A total of 381 patients with PCa from the TCGA cohort and 90 patients from the GEO cohort (GSE70769) were included in the analysis. The baseline demographic and clinical characteristics of the study subjects are shown in Table S2 and S3. To evaluate the expression differences of fatty acid-related genes between tumor tissues and adjacent normal tissues, we analyzed RNA-seq data from the TCGA dataset.

**Figure 1:**
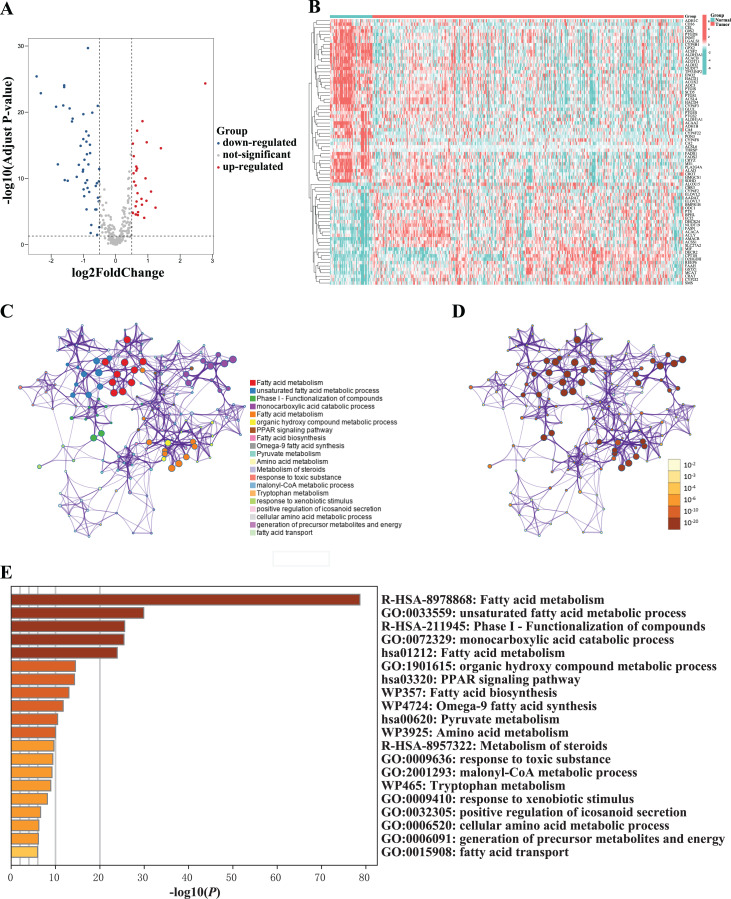
Identification and functional enrichment analysis of differentially expressed fatty acid metabolism-related genes between normal and tumor sample in the TCGA cohort. (A) Volcano plot of differentially expressed fatty acid metabolism-related genes between normal and tumor sample. (B) Heatmap of differentially expressed fatty acid metabolism-related genes between normal and tumor sample. (C–D) Network of enriched terms colored according to (C) cluster ID (nodes with the same cluster ID were typically close to each other) and (D) *p*-value (terms with more genes tended to have higher *p*-values). (E) The top 20 enriched terms of differentially expressed fatty acid metabolism-related genes between normal and tumor sample.

Subsequently, 78 fatty acid-related DEGs were screened out between 381 tumor tissues and 52 adjacent normal tissues, including 29 upregulated and 49 downregulated genes (Figs. 1A and 1B; Table S4). The DEGs between tumor tissues and adjacent normal tissues were analyzed in the TCGA-PCa cohort to clarify their biological functions and pathways. As expected, these fatty acid-related DEGs were significantly related to fatty acid metabolism (Figs. 1C–1E).

### Construction of a fatty acid metabolism-related gene signature

**Figure 2:**
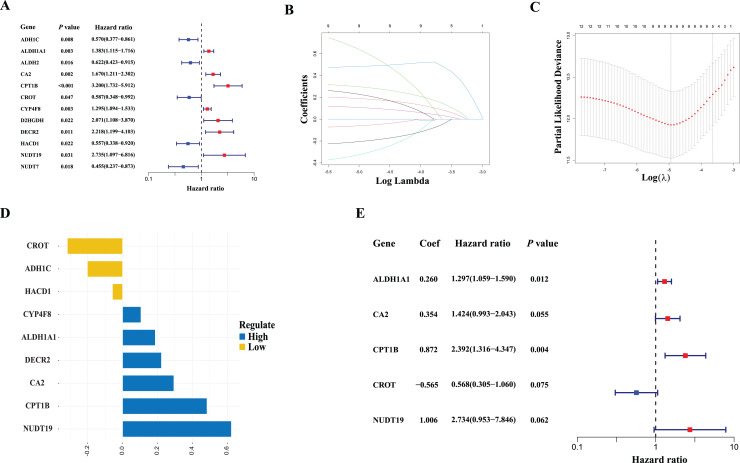
Establishment of the prognostic model in the TCGA cohort. (A) Results of the univariate cox analysis of the biochemical recurrence-free survival in the TCGA cohort. (B) Ten-time cross-validation for tuning parameter selection in the LASSO Cox-regression model in the TCGA cohort. (C) LASSO coefficient profiles of the nine fatty acid-related genes in the TCGA cohort. (D) Coefficients of the LASSO-Cox model with the minimum criteria in the TCGA cohort. (E) Results of multiple stepwise Cox regression of the biochemical recurrence-free survival in the TCGA cohort.

**Figure 3:**
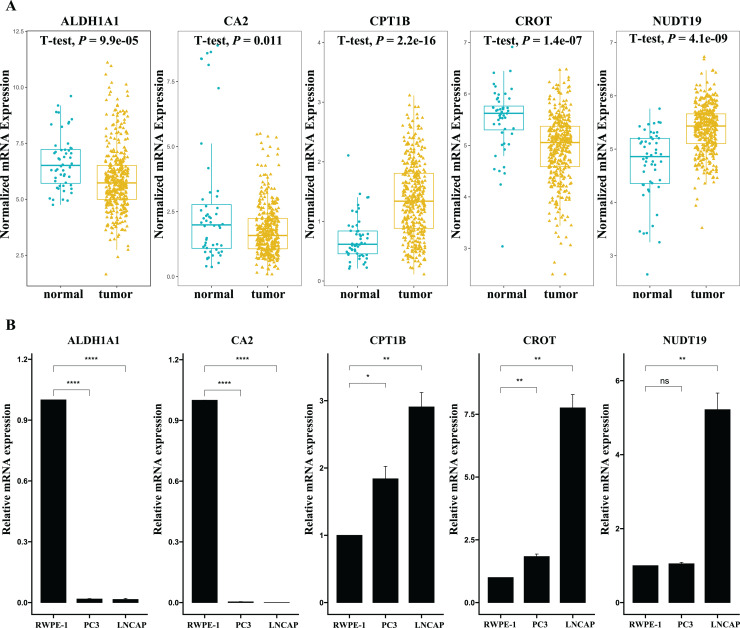
Validation of the mRNA expression levels of the five fatty acid metabolism related-genes in cell lines. (A) Expression of the five fatty acid metabolism-related genes in normal and tumor samples in the TCGA dataset. (Normal samples: 52; Tumor samples: 381) (B) Expression of the five fatty acid metabolism-related genes in two prostate cancer cell lines (PC3 and LNCAP) and normal prostate cell lines (RWPE-1). (Number of experimental repetitions: 3) *P < 0.05, **P < 0.01, ****P < 0.0001, ns: not significant.

Twelve candidate fatty acid-related DEGs associated with BCR-free survival were identified using the univariate Cox regression analysis (Fig. 2A). We then performed LASSO-Cox regression analysis on the identified prognostic DEGs. As shown in Figs. 2B and 2C, using the minimum lambda criteria, nine mRNAs were selected for further analysis. The coefficients of these mRNA are shown in Fig. 2D. To identify the fatty acid-related genes with the greatest prognostic value, we conducted a multiple stepwise Cox regression to construct a risk signature for BCR-free survival prediction, and five genes were selected: *ALDH1A1*, *CA2*, *CPT1B*, *CROT*, and *NUDT19* (Fig. 2E). Further, the expression levels of the five genes were validated in PC3 and LNCaP cell lines by qPCR. As shown in Fig. 3B, the expression levels of *ALDH1A1* and *CA2* were significantly downregulated in PCa cells compared with those in RWPE-1 control cells, while the expression of *CPT1B* and *NUDT19* was higher in PCa cells, consistent with the findings from the TCGA dataset. However, expression of CROT was reversed compared to that in the TCGA database (Fig. 3A); further studies in other PCa cells lines and PCa tissue are needed to confirm these findings.

### Clinicopathological characteristics associated with the 5-gene risk signature

**Figure 4:**
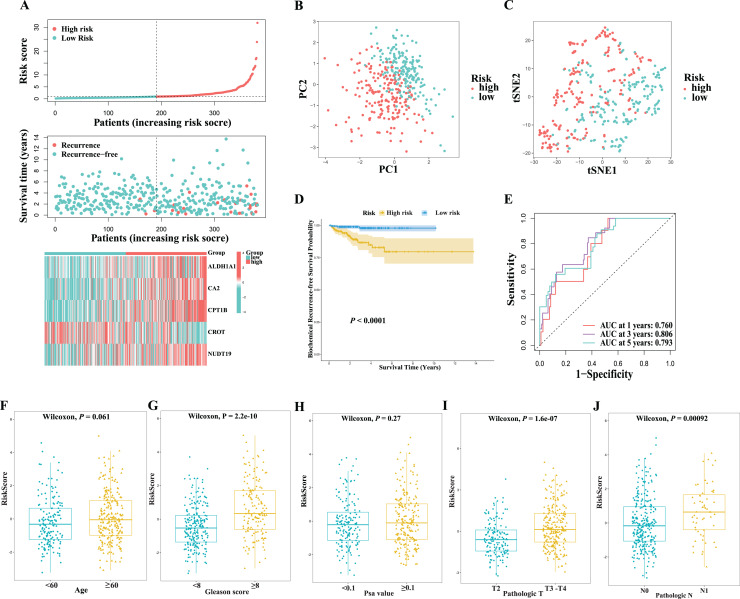
Clincal associations of the fatty acid metabolism-related gene score in prostate cancers from TCGA cohort. (A) The distribution of risk score, biochemical recurrence-free survival status, and the exoression of the five hub genes between the low- and high-risk groups in the TCGA cohort. (B) The PCA analysis of risk score in the TCGA cohort. (C) The t-SNE analysis of risk score in the TCGA cohort. (D) Kaplan–Meier analysis between the low- and high-risk groups in the TCGA cohort. (E) ROC curve and AUC of risk score for predicting the 1/3/5-years survival in the TCGA cohort. (F–J) Risk score by patient age, Gleason score, PSA value, tumor pathological T-stage, and N-stage in the TCGA cohort.

Risk score was calculated for each patient according to the coefficients of selected genes. Patients in the TCGA training cohort were classified as high- (*n* = 190) or low-risk (*n* = 191) based on the median cutoff value of the risk score. As shown in Fig. 4A, patients with high risk scores were more likely to have BCR than those with low risk scores. The results of PCA and t-SNE analyses indicated that patients in the high- and low-risk groups were distributed in discrete directions (Figs. 4B and 4C). Additionally, patients in the high-risk group had poorer BCR-free survival than those with low risk scores (Fig. 4D). The area under the ROC curve for 1-year, 3-year and 5-year BCR-free survival were 0.760, 0.806, and 0.793, respectively (Fig. 4E). Furthermore, as indicated in Figs. 4F–4J, tumors with higher risk score were associate with larger tumor size, more likely to have lymph node involvement, and higher Gleason score, albeit no significant associations with age and prostate-specific antigen (PSA) value were observed. Moreover, multivariate Cox regression analyses showed that the risk score remained independently predictive of BCR-free survival after controlling for major confounders including tumor size, PSA at diagnosis, and Gleason score (HR = 1.11, 95% CI [1.05–1.17], *P* < 0.001, Table 1). In contrast, the Gleason score rendered insignificant after controlling for confounders. These findings suggest that our risk signature might serves as a potential powerful prognostic indicator in guiding clinical practice.

**Table 1:** Univariate and multivariate cox regression for biochemical recurrence-free survival in the TCGA and GEO dataset.

	Univariate analysis		Multivariate analysis*	
	Hazard ratio (95% CI)	*P* value	Hazard ratio (95% CI)	*P* value
TCGA dataset				
Age	1.00 [0.94–1.06]	0.984		
Gleason score	2.40 [1.57–3.69]	<0.001	1.60 [0.99–2.58]	0.057
PSA value	1.10 [1.06–1.15]	<0.001	1.07 [1.02–1.11]	0.002
Pathologic T				
T2	Reference		Reference	
T3-T4	15.27 [2.06–113.07]	0.008	6.97 [0.87–55.70]	0.067
Pathologic N				
N0	Reference			
N1 *vs* N0	1.78 [0.70–4.52]	0.224		
Risk score	1.14 [1.09–1.20]	<0.001	1.11 [1.05–1.17]	<0.001
GEO dataset				
Gleason score	1.94 [1.38–2.71]	<0.001	1.33 [0.89–1.99]	0.164
PSA at diagnosis	1.03 [0.99–1.08]	0.122		
Pathologic T				
T0-T2	Reference		Reference	
T3	4.21 [2.19–8.10]	<0.001	2.98 [1.48–6.00]	0.002
Risk score	2.77 [1.75–4.38]	<0.001	2.05 [1.21–3.47]	0.007

**Note:**

* Significant variables in univariate analysis (*P* < 0.05) were included in multivariate analysis.

### Validation of the 5-gene risk signature

**Figure 5:**
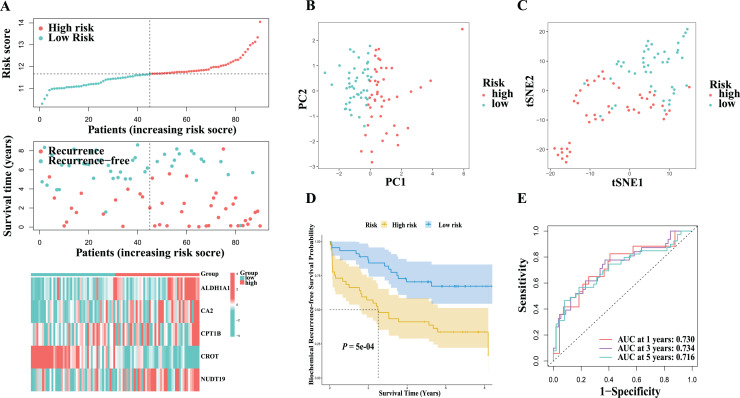
Clincal associations of the fatty acid metabolism-related gene score in prostate cancers from GEO cohort. (A) The distribution of risk score, biochemical recurrence-free survival status, and the exoression of the five hub genes between the low- and high-risk groups in the GEO cohort. (B) The PCA analysis of risk score in the GEO cohort. (C) The t-SNE analysis of risk score in the GEO cohort. (D) Kaplan–Meier analysis between the low- and high-risk groups in the GEO cohort. (E) ROC curve and AUC of risk score for predicting the 1/3/5-years survival in the GEO cohort.

To validate the prognostic significance of the 5-gene risk signature, the corresponding risk score was calculated for each patient in the GEO cohort using the same formula; then, patients were assigned into high- and low-risk group according to the medial value of the score. As shown in Fig. 5A, the expression patterns of the five prognostic genes in GEO cohort were similar to that in the TCGA cohort. Patients with high risk were more likely to have BCR than those with low risk scores. Likewise, the results of PCA and t-SNE analyses indicated that patients in the high- and low-risk groups were distributed in discrete directions (Figs. 5B and 5C). High-risk group also had poorer BCR-free survival compared with those in the low-risk group, consistent with the observation in the TCGA dataset (Fig. 5D). The area under the ROC for 1-year, 3-year, and 5-year BCR-free survival was 0.730, 0.734, and 0.716, respectively (Fig. 5E), comparable to that of the TCGA cohort. Moreover, in line with the findings from the TCGA cohort, the multivariate Cox-regression also revealed that the 5-gene risk score was a robust predictor for BCR-free survival independent of tumor size and Gleason score in the GEO cohort. Taken together, our results demonstrated that our fatty acid metabolism-related 5-gene signature is an independent prognostic predictor of BCR in prostate cancer.

### Association with sensitivity to immunotherapy/chemotherapy

**Figure 6:**
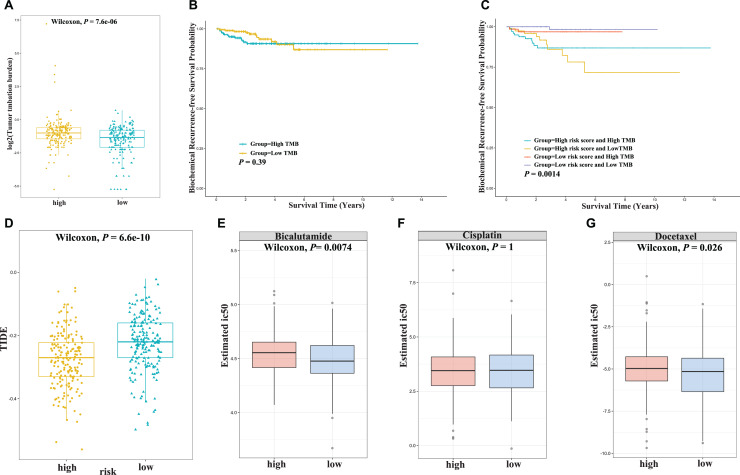
Association with sensitivity to immunotherapy/chemotherapy in the TCGA cohort. (A) Tumor mutation burdern (TMB) between the high-risk and low-risk groups in the TCGA cohort. (B) Kaplan–Meier curve analysis of biochemical recurrence-free survival is shown for patients with high and low TMB values in the TCGA cohort. (C) Kaplan–Meier curve analysis of biochemical recurrence-free survival is shown for patients classified according to the TMB value and fatty acid-related model in the TCGA cohort. (D) Tumor immune dysfunction and exclusion (TIDE) score between the low- and high-risk groups in the TCGA cohort. (E–G) The drug sensitivity of bicalutamide (E), cisplatin (F), and docetaxel (G) between the low- and high-risk groups in the TCGA cohort.

Immunotherapy has been the focus of attention in cancer treatment; however, only a small proportion of patients respond to immunotherapy, highlighting the unmet need of biomarkers for response prediction. TMB is an emerging biomarker for immunotherapy response and has been approved as a companion diagnostic marker for immunotherapy (Marcus et al., 2021). To determine whether the 5-gene risk score could serve as a potential biomarker for immunotherapy, we evaluated its associations with TMB. As shown in Fig. 6A, a higher TMB score was observed in the high-risk group compared to the low-risk group. Although the TMB score alone was not predictive of BCR-free survival, the combination of TMB and risk score could identify a subgroup of patients with high-risk scores and low-TMB scores who had the worst survival (Figs. 6B and 6C). Moreover, significantly lower TIDE scores were observed in the high-risk group than the low-risk group, suggesting a favorable response to immunotherapy associated with a high risk score (Fig. 6D). Next, we determined whether the risk score was also associated with chemotherapy response. The half-maximal inhibitory concentrations (IC_50_) of three commonly used chemotherapeutic drugs (bicalutamide, cisplatin, and docetaxel) in high- and low-risk tumors were compared. As shown in Figs. 6E–6G, high-risk score was related to high IC_50_ values for bicalutamide and docetaxel, which indicates a lower sensitivity to chemotherapy. In summary, the above results suggested that patients with high risk score might benefit from immunotherapy rather than chemotherapy.

### Functional analyses in the TCGA cohort

**Figure 7:**
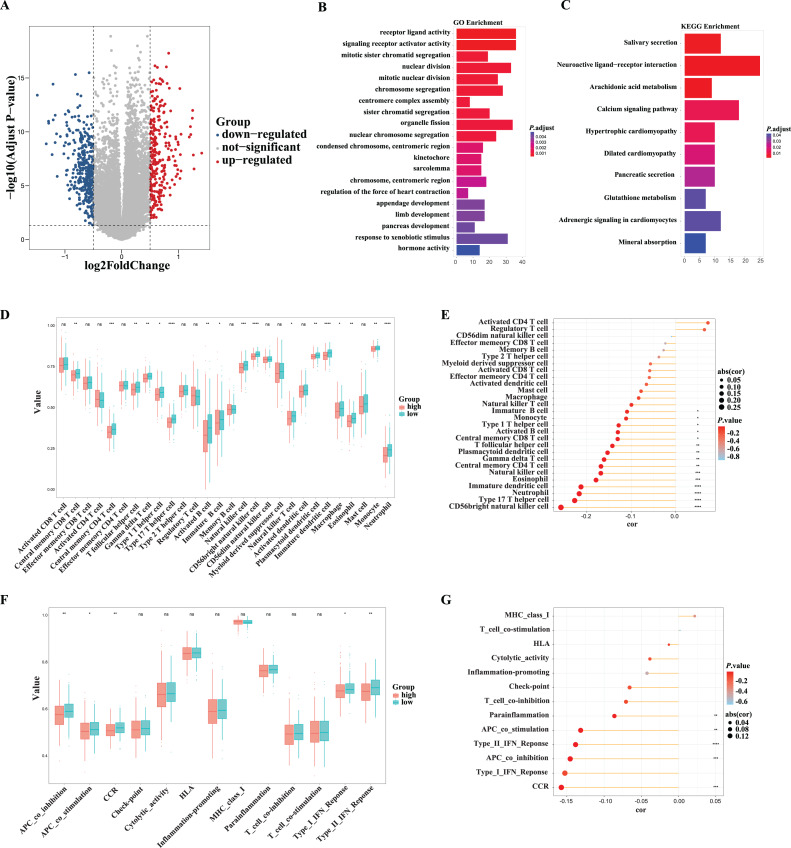
Functional analysis of genes associated with the fatty acid-related risk score in the TCGA cohort. (A) The differentiated expressed genes (DEGs) between the low- and high-risk groups in the TCGA cohort. (B) The GO enrichment of DEGs between the low- and high-risk groups in the TCGA cohort. (C) The KEGG pathway enrichment of DEGs between the low- and high-risk groups in the TCGA cohort. (D, E) Comparison and correlations of the ssGSEA scores of immune cells in the high- and low-risk groups in the TCGA cohort. (F, G) Comparison and correlations of the ssGSEA scores of immune-related functions in the high- and low-risk groups in the TCGA cohort. *P < 0.05, **P < 0.01, ***P < 0.001, ****P < 0.0001, ns: not significant.

Next, we performed enrichment analysis to investigate the potential biological processes underling the fatty acid metabolism-related signature. A total of 637 DEGs between the high- and low-risk groups were identified in the TCGA-PCa cohort, including 279 upregulated and 358 downregulated genes (Fig. 7A; Table S5). GO analysis indicated that these DEGs were enriched in cell division-related biological processes, such as mitotic sister chromatid segregation, nuclear division, mitotic nuclear division, and chromosome segregation (Fig. 7B; Table S6). KEGG functional enrichment analysis suggested that these DEGs were related to salivary secretion, neuroactive ligand-receptor interaction, calcium signaling pathway, and arachidonic acid metabolism (Fig. 7C; Table S7). The immune cells in the TME play important roles in tumor initiation and progression. In this study, distinct tumor immune cell infiltration features were observed between high- and low-risk tumors in the TCGA dataset. Overall, the risk score was inversely associated with abundances of most immune cells, such as CD56^bright^ NK cells, NK cells, central memory CD4^+^ T cells, central memory CD8^+^ T cells, *etc*. (Figs. 7D and 7E). Accordingly, immune activation-related pathways, including antigen-presenting cell (APC) co-inhibition, APC co-stimulation, cytokine–cytokine receptor (CCR), and type II IFN response, were also significantly inversely associated with the risk score (Figs. 7F and 7G), suggesting an immunosuppressive feature in the TME which might contribute to the poor prognosis in high-risk tumors.

## Discussion

In this study, we constructed and validated a prognostic prediction model based on five genes related to fatty acid metabolism for patients with PCa. The risk score proved to be independently predictive of BCR-free survival. Moreover, it was associated with tumor immune infiltration characteristics and could identify tumors with potential sensitivity to immunotherapy but resistance to chemotherapy. Our study may further the understanding of fatty acid metabolism in cancer progression and therapeutic response, providing cues for more effective treatment strategies.

Our risk score demonstrated significant correlations with clinicopathological features, including tumor size, lymph node involvement, and Gleason score. It is worth noting that the PSA value and Gleason score are well recognized biomarkers for PCa and have been incorporated into guidelines for PCa diagnosis, management, and surveillance (Mottet et al., 2021). However, in the current study, the Gleason score rendered insignificant when the risk score was included in the regression model in the TCGA cohort. Also, the PSA value at diagnosis was not predictive of BCR in the GEO cohort. In contrast, our risk score remained significantly predictive of BCR-free survival even after controlling for major confounders, with a more profound effect than the PSA value and Gleason score. Our study suggests that the 5-gene fatty acid metabolism-related risk score might serve as a novel predictive biomarker even if it cannot replace the PSA value and Gleason score.

In our study, the risk model was constructed based on the mRNA expression levels of five fatty acid metabolism-related genes: *ALDH1A1, CA2, CPT1B, CROT, and NUDT19*. Among them, *ALDH1A1* and *CPT1B* were previously shown to be associated with PCa. *ALDH1A1*, as an aldehyde dehydrogenase, can transform precursor retinaldehydes into retinoic acid receptor (RAR) (Kiefer et al., 2012) and has been found to promote PCa invasion and metastasis through the activation of RARα (Jiang et al., 2020). Furthermore, *ALDH1A1*-positive cancer cells exhibited important cancer stem cell properties featured by high *in vitro* tumorigenicity, *in vivo* tumor initiation and self-renewal capacities, and successively reinitiating transplantable tumors (Li et al., 2010). This may explain why high-risk tumors with high *ALDH1A1* expression had poor prognosis. Thus, targeting *ALDH1A1* may be a promising therapeutic strategy. Carnitine palmitoyl transferase 1B encoded by *CPT1B* gene is the rate-limiting enzyme of fatty acid oxidation and regulates intracellular lipid metabolism by translocating long-chain fatty acids from the cytoplasm to the mitochondria for β-oxidation (Britton et al., 1995; Weis et al., 1994). In castration-resistant prostate cancer (CRPC) cells, *CPT1B* overexpression results in upregulated cell proliferation, increased S-phase distribution, and upregulated invasive ability (Abudurexiti et al., 2020). Also, *CPT1B* was reported to be upregulated in prostate cancer and correlated with poor prognosis, in support of our findings (Abudurexiti et al., 2020). However, the roles of *CA2*, *CROT*, and *NUDT19* remain unclear in PCa. Carbonic anhydrase 2 (*CA2*), as a member of the carbonic anhydrase family, is involved in catalyzing the reversible hydration of CO_2_. It has been found that low *CA2* expression in gastric cancer and non-small cell lung cancer promotes tumor proliferation and metastasis. (Chiang et al., 2002; Li et al., 2012). In our study, we also found reduced *CA2* expression in prostate cancer tissues, but whether this promotes tumor progression in prostate cancer remains unknown and needs to be investigated further. Carnitine O-octanoyltransferase (*CROT*) is a peroxisomal enzyme involved in fatty acid β-oxidation (Fernandez-Hernando et al., 2011). Previous studies have reported that *CROT* can be used as a BCR predictor for PCa (Chen et al., 2019), but the biological mechanism remains unclear and further research is warranted to verify these findings. Nudix hydrolase 19 (*NUDT19*) is a peroxisomal enzyme belonging to the nudix hydrolase superfamily of enzymes, which have been linked to the regulation of renal CoA levels *in vivo* (Naquet et al., 2020; Shumar et al., 2018). However, relevant studies in tumors are still scarce, and the exact mechanisms are not yet clear. Overall, the underlying mechanisms of these genes are worth further investigation.

To evaluate the use of our risk score in immunotherapy, we used the TMB and TIDE scores to assess immunotherapy response. TMB refers to the total number of somatic mutations in the tumor genome and has emerged as a predictor of immunotherapy response in cancer (Allgauer et al., 2018; Chan et al., 2019). In this study, TMB values were higher in the high-risk group, indicating that this group may have better immunotherapy outcomes (Yarchoan, Hopkins & Jaffee, 2017). The TIDE score is produced from an algorithm used to assess the ability of immune checkpoints to block the immune response and serves as a surrogate of traditional single biomarkers for immunotherapy response prediction (Jiang et al., 2018). In this study, the high-risk group was associated with lower TIDE scores, which was suggestive of a better response to immunotherapy (Jiang et al., 2018). The low TIDE score in the high-risk tumors was consistent with the TME immune infiltrating features revealed in our study. Jiang et al. (2018) suggested that tumors with high infiltration of cytotoxic T lymphocytes (also known as CD8+ T cells) tended to evade immune surveillance by introducing T cell dysfunction, which was associated with high TIDE scores and poor response to checkpoint inhibitor-based immunotherapy. Consistently, in the current study, we observed a tendency of lower levels of CD8+ T cells in high-risk tumors, which was associated with lower TIDE scores. This result suggests that lower levels of T cell dysfunction might confer enhanced anti-tumor ability of cytotoxic T cells upon immunotherapy. Interestingly, high-risk score tumors tended to be less sensitive to bicalutamide and docetaxel chemotherapy, as suggested by higher IC_50_ values in these patients. Collectively, our findings suggested that patients with high risk scores might benefit more from immunotherapy rather than chemotherapy, providing an alternative strategy for personalized therapy.

There were some limitations in our study. First, our prognostic model was constructed and validated by using retrospective data from public databases. Prospective real-world data are needed to validate its clinical utility. Second, the sample size of this study was small, and larger populations are needed to verify the accuracy of the results. In addition, our research relied heavily on computational analysis; further *in vivo* and *in vitro* experiments are needed to verify our findings.

## Conclusions

We established and validated a fatty acid metabolism-related gene signature based on the expression levels of *ALDH1A1*, *CPT1B*, *CA2*, *CROT*, and *NUDT19*. This signature was a robust prognostic predictor for BCR and could identify a subgroup of patients sensitive to immunotherapy rather than chemotherapy. Our study proposes the 5-gene fatty acid metabolism-related gene signature as a novel tool in guiding PCa therapy.

## Supplemental Information

10.7717/peerj.14854/supp-1 Supplemental Information 1 Supplementary Information. Table S1. The list of fatty acid-related genes. Table S2. Clinical characteristics of patients with prostate cancer in the TCGA cohort. Table S3. Clinical characteristics of patients with prostate cancer in the GEO cohort. Table S4. The table of differentially expressed fatty acid-related genes between tumor and normal cases in the TCGA cohort. Table S5. The table of differentially expressed genes between high- and low-risk subgroups in the TCGA cohort. Table S6. GO enrichment of significant differentially expressed genes between the high- and low-risk subgroups in the TCGA cohort. Table S7. KEGG enrichment of significant differentially expressed genes between the high- and low-risk subgroups in the TCGA cohort.

10.7717/peerj.14854/supp-2 Supplemental Information 2 The raw data of qRT-PCR.

10.7717/peerj.14854/supp-3 Supplemental Information 3 R scripts.

## Additional Information and Declarations

### Data Availability

The following information was supplied regarding data availability: The GDC TCGA Prostate Cancer (PRAD) data is available at UCSC Xena: https://xena.ucsc.edu/welcome-to-ucsc-xena. The data is available at NCBI GEO: GSE70769.
